# Postoperative surveillance after surgery for colorectal liver metastasis: a cross-sectional study

**DOI:** 10.1308/rcsann.2023.0027

**Published:** 2023-05-23

**Authors:** IC Nzenwa, S Pathak, SR Knight, NG Mowbray, D O’Reilly, RP Jones

**Affiliations:** ^1^ University of Liverpool, UK; ^2^ Leeds Teaching Hospitals NHS Trust, UK; ^3^ University of Edinburgh, UK; ^4^ University Hospital of Wales, UK; ^5^ Liverpool University Hospitals NHS Foundation Trust, UK

**Keywords:** Colorectal Neoplasms, General Surgery, Hepatectomy

## Abstract

**Introduction:**

Colorectal liver metastases (CRLM) are associated with a high recurrence rate after surgery. There is paucity of high-quality evidence regarding the nature and overall benefit of surveillance after hepatectomy for CRLM. As part of a broader programme of research, this study aimed to assess current strategies for surveillance after liver resection for CRLM and outline surgeons’ opinions regarding the benefit of postoperative surveillance.

**Methods:**

An online survey was sent to clinicians performing surgery for CRLM at tertiary hepatobiliary centres in the UK.

**Results:**

There were responses from a total of 23 centres (88% response rate); 15/23 centres used standardised surveillance protocols for all patients. Most centres followed patients up at six months, but there is variation in postoperative surveillance at 3, 9, 18 and beyond 60 months. Patient comorbidities, indeterminate findings on imaging, margin status and assessment of recurrence risk were identified as the major factors influencing personalised surveillance strategies. There was clear clinician equipoise regarding the costs and benefits of surveillance.

**Conclusion:**

There is heterogeneity in postoperative follow-up for CRLM in the UK. High-quality prospective studies and randomised trials are necessary to elucidate the value of postoperative surveillance and identify optimal follow-up strategies.

## Introduction

Colorectal cancer (CRC) is the third leading cause of cancer mortality worldwide.^1^ The most frequent cause of death is colorectal liver metastases (CRLM),^2^ which occur in around half of patients with CRC.^3^ Historically, patients with CRLM were offered palliative chemotherapy, which was associated with 5-year survival rates of less than 5%.^4,5^ However, advances in surgical techniques have increased the number of patients with CRLM who are offered surgery and this has increased 5-year survival rates to 30–60%.^4,6,7^

These liver resections are performed with curative intent, but a significant number of patients develop recurrence within two years of the initial hepatectomy.^6^ Nonrandomised evidence suggests that repeat hepatectomy has morbidity and mortality rates comparable with first-time surgery, and may increase survival in patients with recurrence limited to the liver.^6,8,9^ Percutaneous ablation and other local treatments such as stereotactic ablative radiotherapy also offer potential survival benefit in unresectable liver-limited disease.^10^ Thus, surveillance after liver resection for CRLM is performed with the aim of identifying cancer recurrence at a point where treatment options are available.^11^ However, there is a lack of high-level evidence around whether this surveillance offers any benefit in terms of survival or quality of life. Furthermore, there is no clear consensus on the type or frequency of surveillance after hepatectomy for metastatic colorectal disease.

This snapshot study aimed to identify the current strategies for surveillance after liver surgery for CRLM undertaken at tertiary hepatobiliary centres in the UK, and to explore the opinions of practising UK hepatobiliary surgeons on the benefit of postoperative surveillance. This study was performed as part of a package of work supported by Bowel Cancer UK and the Association of Upper Gastrointestinal Surgery of Great Britain and Ireland (AUGIS), investigating clinical practice and establishing an evidence base for the management of patients with CRLM.

## Methods

### Study design

A survey was designed to provide an overview of the services and surveillance strategy in each UK centre performing liver resection for CRLM. Questions were developed to identify the factors that influence surveillance protocols. The survey had four components; first, data were collected on the size and volume of each hepatobiliary unit. A second component assessed the nature of surveillance after liver resection for CRLM at each institution. The third component collected specific data on the protocols and investigations utilised for postoperative surveillance, and the fourth explored the responding surgeons’ perceptions and opinions on the value of surveillance. The complete survey questions are included in the Appendix.

### Survey circulation and data collection

The survey was distributed via electronic mail to all tertiary, hepatobiliary centres in the UK. Data were collected using Google forms. The survey was accessible for a 9-week period between 1 July 2021 and 3 September 2021, and regular reminder emails were sent via the AUGIS and the Great Britain and Ireland Hepato Pancreato Biliary Association (GBIHPBA) to maximise response rates.

### Ethical approval and reporting guideline

The NHS Health Research Authority online tool (http://www.hra-decisiontools.org.uk/research/) was used to assess whether this study should be regarded as research. As there was no change in treatment or patient care and the participants were not randomised to different groups, ethical approval was not required. This study was reported in accordance with the Strengthening the reporting of cohort, cross-sectional and case-control studies in surgery (STROCSS) 2021 guideline (Supplementary Table 1).^12^

## Results

### Respondent and centre demographics

Of the 26 tertiary hepatobiliary centres invited, 23 centres responded to the survey. The majority of the respondents were consultant/attending surgeons. Table 1 displays the characteristics of the hepatobiliary centres. Most centres (*n*=20) had at least four consultant/attending surgeons performing liver resections. Eight centres performed more than 101 liver resections for CRLM per annum (interquartile range 81–100).

**Table 1:** Demographics of the survey respondents and the hepatobiliary centres

Demographic	Number of hepatobiliary centres
Role of respondent	
** **Consultant/attending	20
** **Trainee/Fellow	3
Number of consultant/attending surgeons performing liver resections	
** **2	1
** **3	2
** **4	9
** **>4	11
Total number of liver resections in an average year	
** **21–40	2
** **41–60	1
** **61–80	4
** **81–100	4
** **101–120	3
** **121–140	3
** **>140	6
Number of liver resections for CRLM in an average year	
** **0–10	1
** **11–20	2
** **21–30	0
** **31–40	2
** **41–50	2
** **51–60	2
** **61–80	3
** **81–100	3
>101	8

CRLM = colorectal liver metastases.

### Nature of surveillance protocols

All centres performed an initial postoperative review by the surgical team within six weeks of surgery. The most common objectives for this initial review were wound/symptom assessment and discussion of histological findings. Plans for future surveillance were discussed in the initial follow-up in only eight centres. Fifteen centres reported the use of a standardised postoperative surveillance protocol for all patients. Five centres reported tailoring their surveillance protocol for specific patient groups e.g. those over 80 years old) and three centres designed surveillance protocols based on individual patient factors such as margin status, indeterminate lesions and disappearing lesions.

The team responsible for facilitating postoperative surveillance varied by centre. At ten centres, surveillance was carried out by the liver surgery team only, whereas in the remaining centres, the role of coordinating postoperative surveillance was shared between the centralised cancer coordinator, the primary colorectal cancer team and the medical oncology team. Ongoing surveillance was performed virtually (via telephone or video call) in nine centres. Eight centres adopted a combination of face-to-face and virtual surveillance and five centres conducted face-to-face surveillance only.

### Strategies and investigations for surveillance

**Figure 1:**
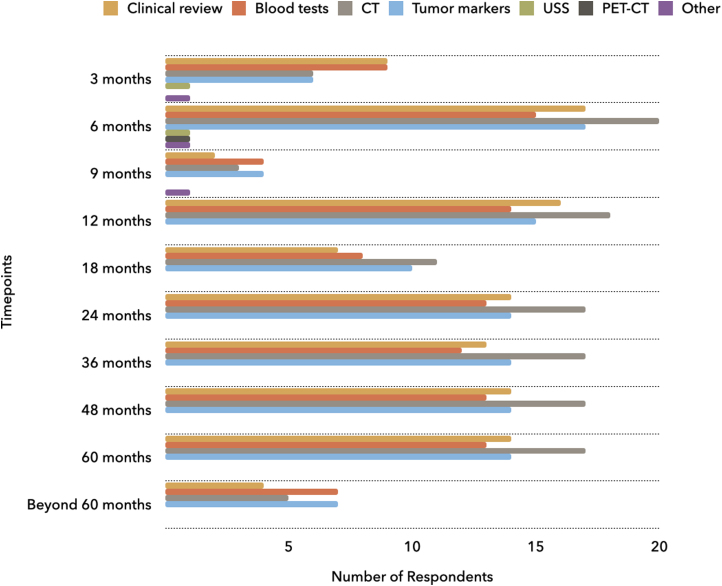
Investigations used for surveillance after surgery for CRLM, including the timepoints at which each modality was used CRLM = colorectal liver metastases; CT = computed tomography; PET-CT = positron emission tomography/computed tomography; USS = ultrasound scan

Figure 1 highlights the variation in the modalities used routinely for postoperative surveillance for CRLM across the UK, including the timepoints at which each investigation was carried out. Computed tomography (CT) scans were the most utilised surveillance modality (*n*=21). Figure 1 shows that almost all centres carried out postoperative surveillance at six months, with most centres also following up at 12, 24, 36, 48 and 60 months. There is a degree of variation between the centres regarding surveillance at 3, 9, 18 and beyond 60 months. Less than one-third of hepatopancreaticobiliary (HPB) centres followed patients up for more than five years. One centre did not report any follow-up strategies.

The respondents identified patient comorbidity (*n*=15), indeterminate findings on scans (*n*=14), resection margin status (*n*=13) and assessment of oncological risk of resected disease (*n*=13) as the most common factors that could influence postoperative surveillance strategies. Less frequently cited factors included clinical symptoms (*n*=9), patient preference (*n*=7), geography/distance to attend surveillance tests (*n*=4) and resource availability (*n*=2).

### Clinicians’ perceptions on the value of surveillance

**Figure 2:**
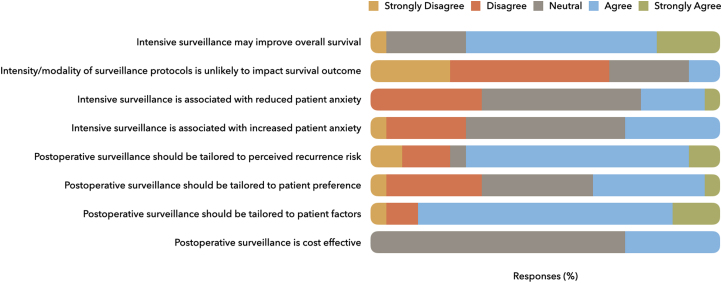
Surgeons’ perceptions regarding postoperative surveillance for CRLM CRLM = colorectal liver metastases

Surgeons’ perceptions of the rationale for postoperative surveillance are represented in Figure 2. Sixteen respondents agreed that overall survival may improve with an intensive surveillance protocol (i.e. shorter surveillance intervals) through an earlier detection of local recurrence and/or earlier initiation of treatment. Conversely, two respondents felt that the intensity or modality of surveillance protocols was unlikely to impact the survival outcome in CRLM. Ten respondents held a neutral position regarding whether an intensive surveillance regime was associated with either increased or decreased patient anxiety.

There were mixed opinions regarding whether surveillance protocols should be tailored to patient preference, as the number of respondents that agreed (*n*=8) and disagreed (*n*=7) with this statement were comparable. Despite these variations, the majority agreed that postoperative surveillance should be tailored to the risk of recurrence (*n*=16) and individual patient factors such as age or comorbidities (*n*=19). Only six respondents agreed that postoperative surveillance protocols are cost effective.

## Discussion

This study demonstrates significant heterogeneity in follow-up after surgery for CRLM in UK hepatobiliary centres. There is variation in several elements, including the time intervals in which surveillance is carried out, the investigations used, the clinicians delivering follow-up, and the general attitude towards follow-up between centres. It has been emphasised that postoperative surveillance aims primarily to detect recurrent disease to ensure the delivery of timely and appropriate interventions, with the ultimate aim of prolonging survival.^6,13^ Although the evidence to support postoperative surveillance is limited, the objective remains to reset the oncological clock by curing, or at a minimum controlling, recurrent metastatic disease.^6^

Based on our findings, the main surveillance modalities used for resected CRLM in the UK were blood tests, CT scans and tumour markers, particularly carcinoembryonic antigen (CEA). Other modalities such as ultrasound scans, magnetic resonance imaging and positron emission tomography scans were seldom used.^14,15^ Most hepatobiliary centres in the UK carried out surveillance until five years after surgery, in line with literature suggestions that the majority of CRLM recurrences occur early.^13,14^

The National Institute for Health and Care Excellence (NICE) recommend a minimum of two CT scans of the chest, abdomen and pelvis in the first three years after colorectal surgery, with regular serum CEA (at least every six months in the first three years) and surveillance colonoscopy a year after the initial treatment.^16^ They suggest further follow-up should be determined by local guidelines, underlining the paucity of evidence in this area.^16^

The Association of Coloproctology of Great Britain and Ireland (ACPGBI) have further evolved these recommendations and now suggest a minimum of two CT scans of the chest, abdomen and pelvis within the first three years of resection (Recommendation grade B) and/or regular serum CEA (every six months in the first three years) (recommendation grade C) with a ‘clean’ colon confirmed by colonoscopy or CT colonography at one year and subsequently at five yearly intervals (Recommendation grade C).^17^ The low levels of recommendation in these guidelines again highlight the lack of compelling data.

The current NICE and ACPGBI guidelines are focused primarily on surveillance after primary colorectal resection, and their applicability to the metastatic setting is unclear. Exploring this gap in the guidance may be a potential catalyst to rapidly advance metastatic disease management in the future. The role of circulating tumour DNA (ctDNA) as a biomarker after curative intent surgery for primary and metastatic colorectal cancer is becoming clearer, with a potential lead-time (over routine scanning and blood tests) of 9 months.^18–20^ With a decrease in cost, a remotely delivered ctDNA surveillance programme that obviates the need for routine scans may become more feasible to pursue.^20^ Although an economic assessment of surveillance strategies was beyond the scope of this study, such a programme is likely to be more cost-effective than unstratified surveillance scanning.

We found that surveillance for CRLM was carried out by multiple specialists including medical oncologists as well as colorectal and liver surgeons. As previously highlighted, the distance to tertiary hepatobiliary centres could have influenced which team primarily oversees patient follow-up. It is also possible that the complexity of CRLM warrants postoperative surveillance that is facilitated by a multidisciplinary team. In addition, postoperative surveillance in some HPB centres has become centrally managed with a nurse-led system. As delivering or coordinating follow-up is not an inherently complex process, the real challenge warranting expert opinion is determining the ideal surveillance strategy for CRLM and the clinical team responsible for its delivery. Organising follow-up for metastatic disease can be difficult as patients are managed simultaneously by different services in different locations, often with different local surveillance policies.

Ignoring the debate around the efficacy of CRLM surveillance, there is a huge potential to optimise time and maximise efficiency through appropriate synchronisation and coordination of surveillance. Decentralised ‘Cancer Navigator’ roles could coordinate a network-wide surveillance programme that ensures scans, colonoscopies and blood tests are performed at the right time and in convenient locations for the patients (e.g. scans in mobile units and blood tests in GP clinics) rather than at a single centralised location. However, this would also require a consensus between specialties regarding what surveillance is needed and when. Though such guidance is theoretically attractive, it would be considerably limited by the relatively poor levels of evidence in this arena.

Our study showed that almost half of the hepatobiliary centres utilised a virtual approach to patient follow-up. The COVID-19 pandemic created a need for alternative routes of communication and patient care.^21,22^ Telemedicine has remarkable potential to improve the efficiency of the current healthcare system and also reduces costs associated with travel, which is particularly relevant for tertiary services where patients may need to travel a significant distance.^21,23^ The limitations and barriers to patients accessing these newer technologies must also be considered. Previous NHS reports have discovered that up to 40% of individuals had no access to online consultations.^21^ These are likely to exacerbate health inequality in patients from lower socioeconomic backgrounds and elderly patients.

The survey highlighted four factors that were deemed most important for influencing surveillance, all of which centred on the perceived risk of recurrence in each patient, as determined by clinical review, radiological or pathological staging. Interestingly, our data reported that most surgeons agreed that surveillance should be tailored to recurrence risk; however, only five centres tailored their surveillance strategies to individual patient risk. A possible explanation for this finding could be that the evidence base supporting additional survival benefit or improved patient outcomes with tailored surveillance protocols is lacking.

There were mixed perceptions regarding whether patient preference should influence surveillance. As with much of the cancer pathway, from diagnosis and treatment to recovery or palliation, there is increased focus on providing a more patient-centred approach, which can be achieved by including patients in the decision-making process.^24^ This entails considering patient preferences when designing surveillance protocols. The role of the patient in the follow-up process has been debated, as increased involvement may lead to patients advocating for more intensive regimes, which could potentially increase anxiety levels.^24^ However, some studies show that the patients’ overall quality of life is unchanged by increased autonomy in the surveillance process.^25,26^ Instead, the reassurance the patients often gain may be highly beneficial.^25,26^ Ultimately, this benefit is best ascertained by investigating patients’ opinions, and future work should assess the impact of postoperative surveillance on patient wellbeing.

There were several limitations to this study. A single individual was asked to respond on behalf of their institution; as such, the response may not be wholly reflective of the views or practice of every clinician/surgeon/oncologist in that centre. The study is also just a snapshot in time and, in light of the aforementioned pandemic, practices are likely to be undergoing review and/or development at present. Finally, five HPB centres tailored their surveillance strategy, but the questionnaire was not able to capture the methods used. The particular strength of this work is the high inclusion rate of UK hepatobiliary centres. By capturing data directly from practising surgeons, the information is up to date and can legitimately be termed expert opinion.

## Conclusion

Currently, there is no consensus for surveillance after hepatectomy for CRLM in the UK. There is heterogeneity in how patients are followed up as well as considerable variation in surgeons’ perception of the benefit of surveillance. Generating evidence for optimal length, frequency and type of surveillance is crucial. A panspecialty agreement on surveillance for patients with metastatic CRC would allow better coordination and efficiency in the postoperative setting. Ultimately, high-quality prospective studies and randomised trials are necessary to elucidate the benefits and rationale for postoperative surveillance.

## Funding

This work was supported by funding from the Association of Upper Gastrointestinal Surgery of UK and Ireland and Bowel Cancer UK.
